# Gender Differences in Childhood Lyme Neuroborreliosis

**DOI:** 10.1155/2015/790762

**Published:** 2015-10-20

**Authors:** Dag Tveitnes, Knut Øymar

**Affiliations:** ^1^Department of Pediatrics, Stavanger University Hospital, Gerd Ragna Bloch Thorsens Gate 8, 4011 Stavanger, Norway; ^2^Department of Clinical Science, University of Bergen, 5020 Bergen, Norway

## Abstract

*Background*. Many neurological diseases show differences between genders. We studied gender differences in childhood Lyme neuroborreliosis (LNB) in an endemic area of Lyme borreliosis in Norway. *Methods*. In a population based study, all children (<14 years of age) with symptoms suspicious of LNB, including all children with acute facial nerve palsy, were evaluated for LNB by medical history, clinical examination, blood tests, and lumbar puncture. LNB was diagnosed according to international criteria. *Results*. 142 children were diagnosed with LNB during 2001–2009. Facial nerve palsy was more common in girls (86%) than in boys (62%) (*p* < 0.001), but headache and/or neck stiffness as the only symptom was more common in boys (30%) than in girls (10%) (*p* = 0.003). The girls were younger than boys and had a shorter duration of symptoms, but boys had a higher level of pleocytosis than girls. In a multivariate analysis, both gender and having headache and neck stiffness were associated with a higher level of pleocytosis. *Conclusion*. Girls and boys have different clinical presentations of LNB, and boys have a higher level of inflammation than girls independent of the clinical presentation.

## 1. Introduction

Lyme borreliosis (LB) is caused by the infection of the spirochete* Borrelia burgdorferi* sensu lato (BB) transmitted to humans during bites of BB infected* Ixodes* ticks. BB has strong invading properties into different human tissues and LB is a multisystem disorder with three clinical time related stages including dermatological, neurological, and rheumatological symptoms [1].

The three main human-pathogen BB genotypes identified in European* Ixodes* ticks,* B. afzelii*,* B. garinii*, and* B. burgdorferi* sensu stricto, are found to have specific tissue preferences in humans.* B. afzelii* is mainly dermatotropic,* B. garinii* is mainly neurotropic, and* B. burgdorferi* sensu stricto is mainly arthrogenic. In US,* B. burgdorferi* sensu stricto is the only genotype identified. However, all organ manifestations may develop in LB infected patients with any of the different BB human-pathogen genotypes [2].

LB is the most frequent tick borne infection in Europe and the northern hemisphere. Erythema migrans (EM), the local and first stage of LB, is the most prevalent LB manifestation [1, 3]. Lyme neuroborreliosis (LNB) develops when the BB spirochete disseminates from the tick bite area and invades and affects the nerve system and is the most frequent second stage manifestation of LB in Europe [1, 4]. The incidence of LNB is higher in children than in adults [3], and, in a European region endemic for LB, LNB was the most common reason for childhood infectious meningitis [5].

In USA, a male predominance has been reported in adult LB [6]. This male predominance has been even more accentuated in children. In an early epidemiological study from the east coast of USA, LB was named the disease of “little boys” [7]. In Europe, with a more variety of BB genotypes, a slightly female predominance has been reported in LB in general and in EM in particular [8, 9]. However, in a recent comprehensive study of Slovenian adults focusing on different organ manifestations, a male predominance was found in Lyme arthritis and LNB [9].

There are few strict population based studies reporting on LNB and gender in children. However, in a comprehensive German study from 1993, the prevalence of LNB in children was twice as high in boys than in girls [10].

South Rogaland, Norway, is an endemic area of LB suitable for epidemiological studies of LNB in children. In population based single centre studies, we have previously reported epidemiological and clinical findings in children with LNB [11, 12], facial nerve palsy (FNP) [13], and Lyme meningitis [5]. The aim of this present study was to study the impact of gender on clinical and laboratory characteristics of LNB in children.

## 2. Material and Methods

### 2.1. Study Area

The coastal area of Rogaland County of the southwest part of Norway is an endemic area of LB, and the incidence of LNB in children is earlier reported among the highest in Europe [11]. The population of South Rogaland, Norway, includes approximately 62 000 children less than 14 years of age, and the Paediatric Department at Stavanger University Hospital receives all hospital admissions for acute childhood disease in this area (with an upper age limit of 14 years).

### 2.2. Patients and Testing Procedures

Prior to the study period, all general practitioners and otorhinolaryngologists in the region received a letter recommending that all children under 14 years of age with acute facial nerve palsy (FNP) should be referred immediately to the Paediatric Department. All children aged 3 months to 14 years during the study period 2001–2009 with symptoms suspicious of LNB, including all children with FNP were evaluated systematically, including medical history, clinical examination, blood tests, and lumbar puncture. In addition, medical records from all children admitted to the hospital during the study period with cerebrospinal fluid (CSF) pleocytosis were identified from the hospital records and studied retrospectively. Data regarding gender, age at diagnosis, year and month of admission, number of days from onset of symptoms to admission, clinical symptoms, and results of blood and CSF tests were registered. Clinical symptoms were considered as not present if not mentioned in the medical record. The study was population based, and only children living in South Rogaland were included.

During the whole study period, in all children with symptoms suspicious of LNB, serum and CSF were analysed for BB IgM antibodies using the Enzygnost Lyme Borreliosis ELISA test (Dade Behring, Siemens, Germany). BB IgG antibodies were measured by the Enzygnost Borrelia IgG test from 2001 to July 2006 and thereafter by Enzygnost LymeLink IgG (Dade Behring, Siemens, Germany). We used the Lyme neuroborreliosis Dako test for detection of intrathecal antibody production defined as antibody index (AI) (Dako, Glostrup, Denmark). Additional microbiological tests were performed when clinically indicated.

White blood cell (WBC) count in CSF was analysed by Adiva 120 (Bayer) or Fuchs Rosenthal chamber (<30 × 10^6^/L) from 2001 to July 2007 and thereafter by XE-5000 (Sysmex). CSF proteins were analysed by Modular (Roche).

The registry data used in this study were anonymous, and therefore a separate ethical license for the study was not obtained. The study was approved by the Privacy Protection Supervisor of the Hospital.

### 2.3. Definitions

Pleocytosis was defined as CSF WBC count >7 × 10^6^/L, according to Clinical and Laboratory Standards Institute guidelines [14].

LNB was diagnosed in children with neurological symptoms suggestive of LNB with the presence of CSF pleocytosis and BB antibodies and/or EM. Confirmed LNB was defined when intrathecal BB antibody production was proven. Probable LNB was defined if BB antibody in serum and/or EM were found in addition to typical symptoms. Definitions were in accordance with the updated European recommendations [15].

FNP was defined as an acute palsy involving the facial muscles in both upper and lower parts of the face, either unilateral or bilateral. Children were diagnosed with headache and/or neck stiffness as the only symptom if they had no other signs of neurological involvement, anorexia or fever.

### 2.4. Statistics

Differences in numbers between groups were analysed by the Chi-square test. Comparisons of continuous data between groups were done by the nonparametric Mann-Whitney *U*-test, and the results are presented as median and quartiles. Variance was tested by Levene's test for equality of variances. The influence of gender and covariates on clinical and laboratory outcomes were analysed by linear regression analyses. Each explanatory variable was first analyzed in simple regression models and in a fully adjusted model. All variables were further included in a backward stepwise multiple regression analyses. The final model included variables significant at the 5% level and interaction was tested for. All analyses were 2 tailed, and data were analysed using the IBM-SPSS statistical package (IBM SPSS Statistics for Windows, Version 22.0, Armonk, NY; IBM Corp.).

## 3. Results

As published earlier, in total, 142 children aged 3 months to 14 years were diagnosed with LNB and included in the study, giving an annual incidence of 26/100 000 [5]. Complete numbers of microbiological tests performed in the children are presented in Table 1. All tests except for BB antibodies were negative.

There was no difference in the total rate between genders, neither for all children nor for children with confirmed or probable LNB (Table 2). However, FNP was more common in girls than in boys with LNB. The total rate of children with headache and neck stiffness did not differ between genders, but headache and/or neck stiffness as the only symptom was more common in boys than in girls (Table 2). Having a history of EM did not differ between genders.

The girls diagnosed with LNB were younger than the boys, but the distribution of age did not differ between genders (Table 2). The girls had a shorter duration of symptoms before admission than boys. The CSF cellular pattern was available in 139/142 children, and 137/139 had predominantly mononuclear cells. All children with LNB were diagnosed from April to December and there was no difference in the monthly distribution between genders.

The median levels of CSF WBC were higher in boys than in girls, whereas the levels of CSF protein and CSF glucose did not differ between genders. Further, the proportion of children with positive BB antibodies in serum or CSF or with a positive BB index did not differ between genders (Table 2).

By univariate linear regression analyses, gender, having facial palsy, or having headache and/or neck stiffness as the only symptom was associated with the level of WBC in CSF, whereas duration of symptoms and age were not associated with the level of WBC (Table 3). In the multivariate analysis, both gender and having headache and neck stiffness remained associated with the level of CSF WBC in the final model (Table 3).

In a multivariate analysis with duration of symptoms before admittance as outcome, FNP was associated with the duration of symptoms (*B* = −14.5 (−18.9, −10), *p* < 0.001), whereas gender and headache and neck stiffness were not associated with the duration of symptoms (data not shown), meaning that gender did not have an independent effect on the duration of symptoms before admittance.

## 4. Discussion

In this single centre population based study which is among the largest studies of childhood LNB in Europe, we found genders differences in childhood LNB not reported earlier. There was no difference in the total incidence between genders, but there were significant differences in the clinical presentation of LNB between girls and boys. A striking finding was that girls more often had FNP than boys, whereas boys more often had headache and/or neck stiffness as the only symptom. Boys had a higher level of inflammation in CSF, and gender and headache and neck stiffness were associated with the level of CSF WBC in a multivariate regression analyses. Girls were younger and had a shorter duration of symptoms before admission, but only FNP was associated with the duration of symptoms before admission in the multivariate regression analyses.

The epidemiological results and conclusions in our study depend on a correct diagnosis of LNB. We based the diagnosis on the most updated European case definitions [15]. According to these case definitions, demonstration of CSF pleocytosis is mandatory for the diagnosis of LNB.

In contrast to our results, several other studies report LNB to be more common in boys than in girls [3, 10, 16, 17]. A male predominance in LNB was recently reported in a comprehensive study in adult patients from Slovenia, where 61% of patients with LNB were male [9]. In a Swedish study of patients of all ages, 62% were male; however, the proportion among children was not specified [18].

Compared to other studies, we found a higher proportion of girls with LNB and they had FNP more commonly. The female predominance of FNP in children with LNB in the present study has not been reported previously to our knowledge. The opposite was reported in a small prospective German study, with 11 of 16 consecutive children with FNP and LNB being boys [19].

We have previously reported the highest incidence of FNP in children in Europe [5]. Children in the study area with FNP were admitted to the Paediatric Department without delay, and a lumbar puncture was performed. To our knowledge, no other studies have used this diagnostic approach [17]. The need for a lumbar puncture for the diagnosis of LNB in children with FNP is still under debate [20, 21]. However, depending on serological blood tests only, high numbers of false negative tests are likely [11, 22]. With our diagnostic routine, we consider the number of children diagnosed with LNB to be close to the true incidence.

In general, LNB is more common in children than in adults, and the frequency of FNP is more common in children than in adult patients with LNB in Europe [3, 23]. Furthermore, Berglund et al. observed that children more often had tick bites in the head and neck region compared to adults, possibly explaining these findings [3]. This may relate to the tick behaviour, questing on average 50 cm above the ground [24]. When playing, children may expose their head and neck to grass and bushes in a different way than adults, and a tick bite in this region may have a higher tendency to result in FNP. Possibly, due to longer hair, girls may be more exposed to BB by catching up questing ticks and thereafter cover the ticks more effectively than in boys with short hair. This may contribute to a higher proportion of girls presenting with FNP due to LNB, but this needs to be further studied. If LNB with FNP is more common in girls as our results suggest, our aggressive approach to children with FNP may have included more girls with LBN compared to other studies, thereby equalizing the male predominance in children with LNB found in other studies.

Headache and/or neck stiffness as the only symptom with absence of other neurological signs has also been shown by others to be a common presentation of childhood LNB [17], which may represent symptoms of meningeal inflammation. This is the first study to show a male predominance in this clinical subgroup of childhood LNB, but the reason for this is not known. Different genospecies of BB may give different clinical presentations of LNB, and all of the human pathogenetic BB gene-species have been detected in questing ticks from a nearby area to the study area [25]. Strle and his group have reported a male predominance in* B. garinii* skin infections [9]. Furthermore, different gene-species of BB are reported to give different clinical presentations of LNB as demonstrated in a study of adult patients with LNB [2]. In that study,* B. garinii* was more common in CSF in patients with typical early LNB with painful meningoradiculoneuritis (Bannwarth syndrome), whereas* B. afzelii* was more common in patients with less specific manifestations of LNB. A speculation could be that the difference in clinical presentation between girls and boys in our study could to some extent be due to different gender disposition for infection with different BB genospecies. However, it has not yet been studied if different genospecies of BB have any gender preferences in children with LNB.

We also found a higher level of CSF inflammation in boys, independent of the clinical presentation. BB produces no toxins and the manifestations of LB and LNB are probably mainly a result of the immune response of the host [26, 27]. In general, the immune system may show differences between genders on a DNA sequence level and on a secondary epigenetic genome regulation level [28]. Furthermore, specific immune responses differ between genders with age, as a result of changing levels of sex steroid hormones through the dynamic interaction with the immune system [29]. Possibly, this could partly explain the observed sex-specific infection rates in humans, even in LNB. In postmenopausal women, a T-helper lymphocyte type 2 (Th2) pattern was associated with a higher tendency for reinfection with borreliosis, compared to men [30]. Also, the CSF cytokine pattern beyond the acute stage of LNB is associated with a Th2 response [31]. Atopy is considered to be a Th2-driven immunopathy, and during childhood atopic disease is more common in boys. We could speculate if this may be related to a more generalised and stronger immune response in boys with LNB, but this has to be further studied.

Further, headache and/or neck stiffness as the only clinical presentation was also independently related to the level of CSF inflammation, suggesting a pathophysiological link. It has been speculated that the dissemination of BB in LNB may differ between meningoradiculitis and more diffuse meningitis [4]. Both Bannwarth's syndrome and FNP may be the result of a local invasion of the spirochete in the nerve root, whereas general meningitis may be the result of haematogenous spread [4, 32]. This may result in differences both in clinical presentation and in laboratory results, as shown in our study. How this is related to gender needs further studies.

The retrospective design of the study may to some extent weaken the quality of the clinical characteristics recorded in the medical records. Data about clinical symptoms are based on parents' and physicians' observations, and some of these variables may be less reliable in younger children. However, we focused on objective variables as FNP, and laboratory results are independent of the retrospective design. It is in our opinion a strength of the study that uniform and extensive procedures to identify and diagnose children with LNB were introduced in the region before the study period and were performed consistently during this period.

## 5. Conclusion

In this single centre population based study of children with LNB, we found that FNP was a more common presentation in girls, whereas headache and/or neck stiffness as the only symptom was more common in boys. Boys had a higher level of CSF inflammation, independent of the clinical presentation. The interplay between the properties of ticks and BB genospecies and children causing Lyme borreliosis is complex. The observed differences between genders may be of importance for the understanding of the pathophysiology in LNB.

## Figures and Tables

**Table 1:** Numbers of positive and negative microbiological tests in 142 children with Lyme neuroborreliosis.

	Number	CSF bacterial culture^*∗*^	BB antibodies	BB antibody index	HSV1 CSF-PCR	Enterovirus CSF-PCR	VZV CSF-PCR	*M. pneumoniae* CSF-PCR	*M. pneumoniae* CF test	EBV-antibody	CMV antibody
*LNB*	**142**	**0/142**	**139/3**	**91/44**	**0/34**	**0/26**	**0/14**	**0/22**	**0/54**	**0/37**	**0/39**
Confirmed NB	91	0/91	91/0	91/0	0/21	0/16	0/10	0/16	0/38	0/27	0/30
Probable NB	51	0/51	48/3	0/44	0/13	0/10	0/4	0/6	0/16	0/10	0/9

^*∗*^Positive/negative tests. LNB: Lyme neuroborreliosis, CSF: cerebrospinal fluid, BB: *Borrelia burgdorferi*, HSV1: herpes simplex virus type 1, PCR: polymerase chain reaction, VZV: varicella zoster virus, *M. pneumoniae*: *Mycoplasma pneumoniae*, EBV: Epstein-Barr virus, CMV: cytomegalovirus, and CF: complement fixation.

**Table 2:** Differences in numbers and clinical and laboratorial characteristics between boys and girls in 142 children hospitalized with Lyme neuroborreliosis (LNB) at Stavanger University Hospital during 2001–2009.

	All children (*n* = 142)	Boys (*n* = 73)	Girls (*n* = 69)	Boys versus girls (*p* value)
All children with LNB (%)		73 (51)	69 (49)	0.89
Confirmed LNB, *n* (%)	91 (64)	46 (63)	45 (65)	0.86
Probable LNB, *n* (%)	51 (36)	27 (37)	24 (35)	0.78
Acute facial palsy, *n* (%)	104 (73)	45 (62)	59 (86)	**0.001**
Headache and neck stiffness; *n* (%)	33 (23)	21 (28)	12 (17)	0.11
Headache and/or neck stiffness as the only symptom *n* (%)	29 (20)	22 (30)	7 (10)	**0.003**
History of erythema migrans, *n* (%)	33 (23)	15 (21)	18 (26)	0.44
Age (years), median (IQR)	6 (5–8)	7 (5–9)	6 (4–7)	**0.005**
Age, variance				0.2
Month of occurrence, variance				0.84
Days of symptoms on admission, median (IQR)	5 (2–14)	7 (3–16)	4 (1–10)	**0.008**
CSF WBC count × 10^6^/L, median (IQR)	166 (90–340)	202 (101–430)	131 (77–280)	**0.004**
CSF protein, mg/L, median (IQR)	520 (340–755)	560 (385–795)	480 (330–700)	0.083
CSF glucose, mmol/L, median (IQR)	3.0 (2.8–3.4)	3.0 (2.8–3.3)	3.1 (2.8–3.4)	0.57
Pos BB Ab^*∗*^ *n*; BB index/s-BB IgM/s-BB IgG	91/113/85	46/58/42	45/55/43	0.86/0.86/0.77

^*∗*^BB: *Borrelia burgdorferi*. 2 girls and 1 boy had ECM but negative BB Ab tests and therefore were diagnosed as probable LNB.

**Table 3:** Linear regression model with cerebrospinal fluid white blood count as dependent variable in 142 children hospitalized with Lyme neuroborreliosis at Stavanger University Hospital during 2001–2009.

Risk factor	Unadjusted model			Adjusted model			Final model		
	*B*	95% CI	*p* value	*B*	95% CI	*p* value	*B*	95% CI	*p* value
Boys	98.1	(34.7, 162)	0.003	73.7	(8.9, 139)	0.021	84.8	(22.7, 147)	0.008
Facial palsy	−117	(−188, −45.5)	0.002	−83.1	(−167, 1.2)	0.053			
Headache and neck stiffness	131	(56.3, 205)	<0.001	99.8	(23.3, 176)	0.011	117	(43.8, 191)	0.002
Age (months)	5.2	(−7.1, 17.4)	0.404	−1.2	(−13.1, 10.8)	0.847			
Duration (days)	1.4	(−1.1, 3.9)	0.27	−1.1	(−3.8, 1.6)	0.412			

There was no interaction between gender and headache and neck stiffness.
